# Improved outcome in penile cancer with radiologically enhanced stratification protocol for lymph node staging procedures: a study in 316 inguinal basins with a mean follow-up of 5 years

**DOI:** 10.1186/s12894-023-01303-9

**Published:** 2023-08-15

**Authors:** Vivekanandan Kumar, Prakrit R Kumar, Arne Juette, Davina Pawaroo, Richard Y Ball, Krishna K Sethia

**Affiliations:** Department of Urology, Norfolk and Norwich University Hospitals NHS Trust, Norwich, NR4 7UY UK

**Keywords:** Inguinal, Imaging, Lymph node, Penile cancer, Sentinel node, Staging

## Abstract

**Background:**

Lymph node metastasis is the main determinant of survival in penile cancer patients. Conventionally clinical palpability is used to stratify patients to Inguinal Lymph node dissection (ILND) if clinically node positive (cN +) or Dynamic sentinel node biopsy (DSNB) if clinically node negative (cN0). Studies suggest a false negative rate (FNR) of around 10% (5–13%) for DSNB. To our knowledge there are no studies reporting harder end point of survival and outcomes of all clinically node positive (cN +) patients. We present our outcome data of all patients with penile cancer including false negative rates and survival in both DSNB and ILND groups.

**Methods:**

One hundred fifty-eight consecutive patients (316 inguinal basins), who had lymph node surgery for penile cancer in a tertiary referral centre from Jan 2008 to 2018, were included in the study. All patients underwent ultrasound (US) ± fine needle aspiration cytology (FNAC) and then MRI/ CT, if needed, to stage their disease. We used combined clinical and radiological criteria (node size, architecture loss, irregular margins) to stratify patients to DSNB vs ILND as opposed to clinical palpability alone.

**Results:**

11.2% i.e., 27/241 inguinal basins had lymph node positive disease by DSNB. 54.9% i.e., 39/71 inguinal basins (IBs) had lymph node-positive disease by ILND. 4 inguinal basins with no tracer uptake in sentinel node scans are being monitored at patient’s request and have not had any recurrences to date.

With a mean follow-up of 65 months (range 24–150), the false-negative rate (FNR) for DSNB is 0%. Judicious uses of cross-sectional imaging necessitated ILND in 2 inguinal basins with non-palpable nodes and negative US with false positive rate of 6.3% (2/32) for ILND. The same cohort of DSNB patients might have had 11.1% (3/27) FNR if only palpability criteria was used. 43 (28%) patients who did require cross sectional imaging as per our criteria had a low node positive rate of 4.7% (*p* = 0.03). Mean cancer specific survival of all node-positive patients was 105 months.

**Conclusion:**

The performance of DSNB improved with enhanced radiological stratification of patients to either DSNB or ILND. We for the first time report the comprehensive outcome of all lymph node staging procedures in penile cancer.

## Background

Lymph node (LN) metastasis is the single most important prognostic factor in patients with squamous cell carcinoma of the penis (SCCp) [1]. Patients with pathologically node-negative (pN0) disease have been reported to have excellent long-term survival. It has also been established that clinically node-negative (cN0) patients have a 25% chance of occult metastases in lymph nodes [2]. Hence the European Association of Urology (EAU) guidelines recommend the use of dynamic sentinel node biopsy (DSNB) in patients with intermediate and high-risk cN0 disease.

The use of DSNB in penile cancer has evolved over the years. Ultrasound of the inguinal basin, with or without fine needle aspiration cytology (FNAC), before DSNB improves the sensitivity of DSNB and reduces false negative rates [3]. Inguinal basins with impalpable LNs and normal ultrasound (US) imaging are defined as cN0. As inguinal basins with palpable LNs may harbour metastasis in up to 70% of cases, ILND would be a safe way of dealing with these cases. However, in view of the high morbidity associated with ILND [4, 5], patients with palpable lymph nodes and subsequent negative US + FNAC are sometimes classified as being cN0. With this approach, the false negative rate (FNR) of DSNB varies from 5–13% in large-scale, short-term follow-up studies [6–9]. None of the studies report on survival of the patients who had undergone DSNB. Only one study reports on harder end point of survival in false negative patients with 100% mortality in them.

The initial stratification of patients into palpable and impalpable lymph node disease is subjective. Factors that influence the palpability of nodes include the clinical experience of the surgeon, the patient’s body habitus, previous inguinal surgery, the presence of infection or other reactive changes, and the consistency of the enlarged lymph node in relation to surrounding tissues. The transition of a lymph node from impalpable to palpable is a gradual process; using palpability as a first definitive risk stratification process is too simplistic in modern practice with excellent cross-sectional imaging capabilities. Further, we suspect that the false-negative rate of approximately 10% in published series is likely related to this simplistic approach.

As a result of such considerations, in our centre we have used radiologically enhanced criteria to stratify patients into cN0 and cN + disease per inguinal basin. All cN0 inguinal basins proceeded to DSNB and cN + to ILND.

This study has two aims: (1) To assess if our radiologically enhanced criteria have better diagnostic performance in sub-stratification of patients into cN0 and cN + ; and (2) To report for the first-time harder end point of survival in our cohort of newly diagnosed SCCp patients, who underwent either ILND and/or DSNB as per our radiologically enhanced stratification criteria.

## Methods

### Patient selection

**Fig. 1:**
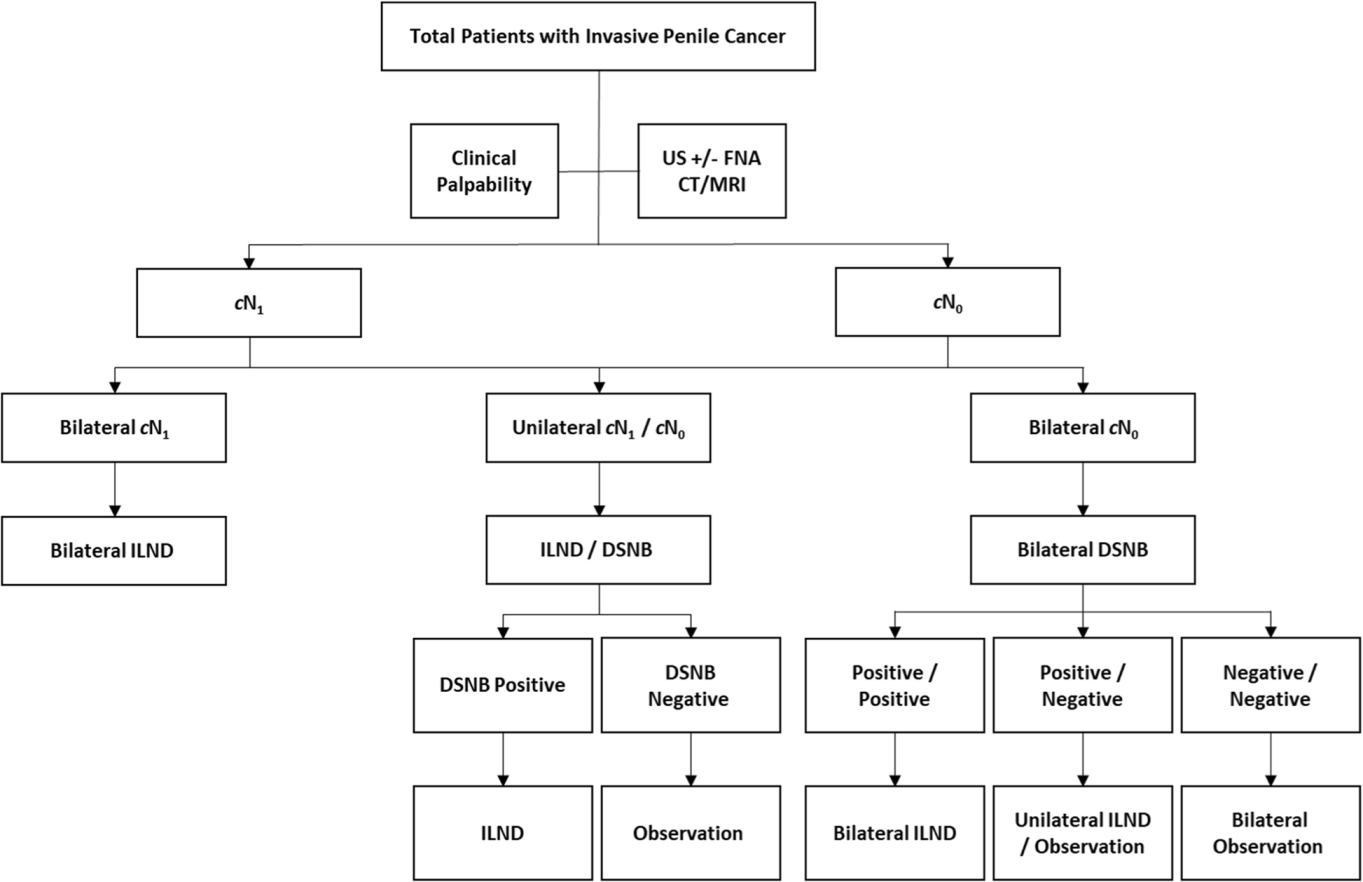
Flow chart of nodal stratification for surgery

All patients who had been referred to our supraregional penile cancer network centre with invasive penile cancer and treated with curative intent were enrolled into this study. Patients were stratified initially on the clinical palpability and US findings. At US, the lymph nodes were graded between U1 and U5 by two experienced radiologists who specialise in axillary and inguinal US (Table 1). All inguinal basins with impalpable nodes and U1-2 LN underwent DSNB. All U3-5 LN had FNAC. If the FNAC was positive for metastasis, they were classified as cN + and had ILND. All patients with U3-5 LN and negative FNAC had CT/MRI scan. The lymph nodes were carefully assessed and discussed at the multidisciplinary team (MDT) meeting. We performed MRI if penile and LN staging were needed together and CT if only LN staging were needed. If the scans showed abnormal features (size ≥ 10 mm, multiple nodes, heterogeneity, irregular margins, and/or loss of fatty hilum) as agreed by the MDT, the patient was recommended to have ILND on that side, despite negative FNAC, as opposed to other protocols which use only palpability criteria (Fig. 1). Hence, some patients with non-palpable lymph nodes and US FNAC-negative had ILND due to liberal availability of low-morbidity Video-endoscopic inguinal lymph node dissection (VEILND) in our centre.

**Table 1:** Ultrasound grading system for inguinal lymph nodes^a^

^a^The grading is based on size, shape, short-long axis ratio, eccentric cortical hypertrophy, absence of an echogenic hilum, hypo-echogenicity of the lymph node, lymph node necrosis and abnormal vascularity using power Doppler
1 = normal
2 = abnormal but benign
3 = abnormal and indeterminate, but with a low risk of malignancy – further assessment required
4 = suspicious with a moderate risk of malignancy – further assessment required
5 = suspicious with a high risk of malignancy – further assessment required (clinical and imaging, path is definitive)

We performed DSNB on all (Low, intermediate, and high risk disease) patients with clinically invasive SCCp, at the time of primary surgery to save multiple general anaesthetics. Hence some low-risk patients also had DSNB as they were clinically (without biopsy) thought to be higher risk category.

We defined false-negative (FN) DSNB as the development of regional nodal recurrence after a negative DSNB, without evidence of a new primary tumour or SCCp recurrence that predated nodal recurrence. FNR is calculated by formula FN/FN + TP (True Positive).

We defined false positive (FP) for ILND if the nodes are negative (pN-) for metastasis when they were clinically node positive (cN +) by our stratification criteria. False positive rate for ILND is calculated by formula FP/FP + TN (true negative).

### DSNB technique

All patients with invasive penile cancer underwent DSNB as per the modified technique reported by Leijte et al., which is considered the ‘gold standard’ for DSNB [10]. This involves preoperative lymphoscintigraphy on the day of surgery and marking over the nodes on the skin, injection of blue dye for intraoperative visualisation, preoperative US _ FNAC, intraoperative identification of radioactivity using a c-probe, and removal of any palpable abnormal feeling nodes during surgery. We used planar imaging till 2014 and SPECT-CT as a part of DSNB after that to identify the sentinel nodes.

### Open and (VEILND) Technique

The technique has been reported in our previous lymphadenectomy series [11]. The VEILND procedure was carried out as a 3-port technique, and patients were left with indwelling tube drain for 1–3 weeks. Open ILND is performed as the same template as VEILND with an S-shaped incision across the inguinal fold. All the ILND had been performed by a single high-volume surgeon in a tertiary supra-network referral centre. DSNB was done by two high volume surgeons in the same tertiary referral centre and we have perform just over 30 procedures each year.

### Histopathology

All sentinel lymph nodes (SLN) and associated LNs were serially sliced at about 2 mm intervals, processed and completely embedded as paraffin wax blocks. Haematoxylin and eosin (H&E)-stained sections were examined by an experienced histopathologist. At least one representative block of all negative SLNs underwent immunohistochemical staining (IHC) for keratins to exclude unseen single-cell metastatic or micrometastatic disease.

### Follow-up

All patients in the study had clinical examination every 3 months in the first year, every 4 months in the second year, and every 6 months for a further 3 years, at the tertiary centre by the surgeon performing the lymph node staging procedure. All DSNB-negative patients had inguinal ultrasound every 3–6 months to identify any enlarged nodes early. If nodes were found during follow-up, US FNAC was performed if the nodes were graded U3-5. If patients had persistently enlarged lymph nodes despite negative FNAC, they proceeded to trucut biopsy or open excision biopsy. Node-positive patients also had CT during follow-up if they were obese, and/or had high-risk disease at the discretion of the surgeon. Further pelvic nodal surgery and adjuvant therapy were considered, determined by the number of positive nodes and the presence of extra-nodal disease in the ILND specimen.

### Data analysis

All data were collected prospectively in an institutional database as per Trust information governance protocol and analysed using SPSS Statistics (IBM, SPSS, V26). Continuous variables were summarized using the mean and standard deviation. Categorical data were summarized using count and percentages. We report all the results as per patient and per inguinal basin, with the % reported as per patient, which is clinically relevant. Sensitivity and false negative rates were calculated per patient or per inguinal basin, as needed. The chi-squared test was used for binary and categorical variables, with *p* < 0.05 considered as statistically significant. Kaplan–Meier curves were constructed using SPSS, and log rank test was used to compare significance of difference in survival between the two groups.

## Results

**Fig. 2:**
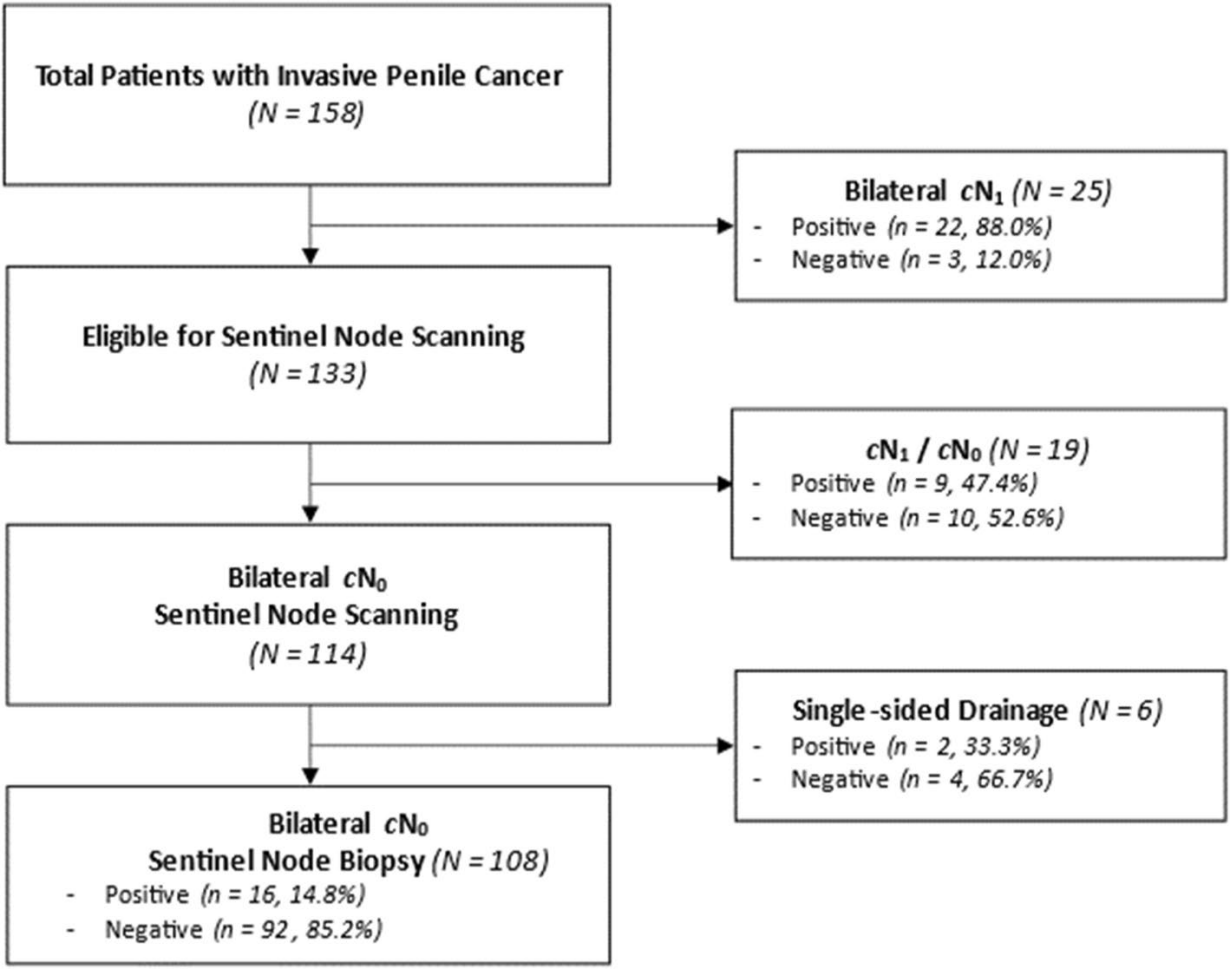
Flow chart of patient recruitment

A total of 158 patients were treated for penile cancer with curative intent from 2008 to 2018 at our institution (Fig. 2). Twenty-five patients (mean age = 68 years), who had bilateral cN + disease, underwent bilateral radical ILND. One hundred-eight patients (mean age = 67) with bilateral cN0 disease had bilateral DSNB. 19 patients with unilateral cN0 disease (mean age = 67 years) underwent DSNB on that side and ILND in the other side.

Two hundred forty-one IBs had DSNB due to clinically node negative disease(cN0) and 71 IBs had ILND due to abnormal nodes (cN +) and 4 IBs were observed due to non-drainage in sentinel node scan. 27(11.2%), 39 (54.9%) and 0 of those had nodes positive for metastasis respectively. The minimum and mean follow-up is 28 and 65 months respectively. FNR is 0%.

The mean number of sentinel nodes removed were 2.32 and 2.52 (range 1–5) in the right and left respectively. Mean number of lymph nodes positive was 1.

Six patients had unilateral tracer uptake during lymphoscintigraphy, which gives a visualisation rate of 95.5% per patient and 97.7% per inguinal basin. All 6 patients were offered ipsilateral prophylactic ILND. Of these, two patients had ILND (one immediate and one delayed) and four patients opted for active surveillance. One of these ILND patients had node-positive disease (16.7%). All six of these patients have remained disease-free.

The grading and staging details of the primary tumours are shown in Table 2. Of the T1 patients 10 had pT1a disease. 38% of stage T3 patients had lymph node metastasis compared to 15.2% of T1-2 patients (*p* = 0.003). 30% of G3 patients had lymph node metastasis compared to 8% of G2 patients (*p* = 0.03) (Table 3).

**Table 2:** Tumour grade and stage characteristics of patients undergoing DSNB (%)

Stage	G1	G2	G3	Total
T1	10 (7.5)	36 (27.1)	28 (21.1)	74 (55.6)
T2	3 (2.3)	10 (7.5)	25 (18.8)	38 (28.6)
T3	0 (0)	4 (3)	17 (12.8)	21 (15.8)
Total	13 (9.8)	50 (37.6)	70 (52.6)	133

**Table 3:** DSNB-positive status as per risk category, grade and T stage

Parameter	Total number of patients	Number of patients with positive LN(s)	% of patients with positive LN(s)
Intermediate risk	36	4	11.1
High risk	87	21	24.1
T1	74	11	14.9
T2	38	6	15.8
T3	21	8	38.1
G2	50	4	8.0
G3	70	21	30.0

One hundred eleven out of one hundred fifty-eight patients had cross-sectional imaging. 42/44 patients (96%) who had ILND had cross sectional imaging. 22 had CT, 18 MRI and 2 had both. 2 and 3 IBs which had impalpable nodes, but cN + by radiological criteria, were pathologically node negative and pathologically node positive respectively. 65/114 cN0 group patients had cross-sectional imaging. 43 patients of cN0 group who had normal ultrasound (U1-2) and impalpable nodes did not have any cross-sectional imaging (Table 4). Interestingly, only 2/43 (4.7%) patients who did not have cross-sectional imaging were DSNB-positive for metastatic disease as opposed to 14/65 (21.5%) of imaged patients, which was statistically significant (*p* = 0.03).

**Table 4:** Cross-sectional imaging data in different groups of patients

Imaging	Bilateral DSNB	Bilateral ILND	Unilateral DSNB/ ILND	Single-sided drainage	Total
CT	30	13	9	1	53
MRI	34	11	7	3	55
Both	1	1	1	0	3
None	43	0	2	2	47
Sum	108	25	19	6	158

The mean follow-up period was 65 months (range 24 to 150). So far, none of the patients who had DSNB surgery has had inguinal lymph node recurrence, which makes the false negative rate 0% (Table 5).

**Table 5:** Summary of disease characteristics of patients with non-palpable nodes who are true and false positives

Study number	Age	Grade	Stage	Growth pattern	LVI	SCC Type	Primary treatment	Margin status	Number of nodes excised	Number of positive nodes	Complications
Non palpable nodes, but True positives											
58	69	3	1	endophytic	yes	basaloid	Circumcision	negative	9	1	none
81	82	3	3	endophytic	yes	basaloid	Glansectomy	negative	7	1	none
88	77	2	1b	endophytic	yes	usual	Partial penectomy	negative	16	1	seroma
Non palpable nodes, but False positives											
101	87	Breslow 4	3	endophytic	yes	melanoma	Glansectomy	negative	8	0	none
127	53	2	2	exophytic	no	usual	Glansectomy	negative	7	0	none

**Fig. 3:**
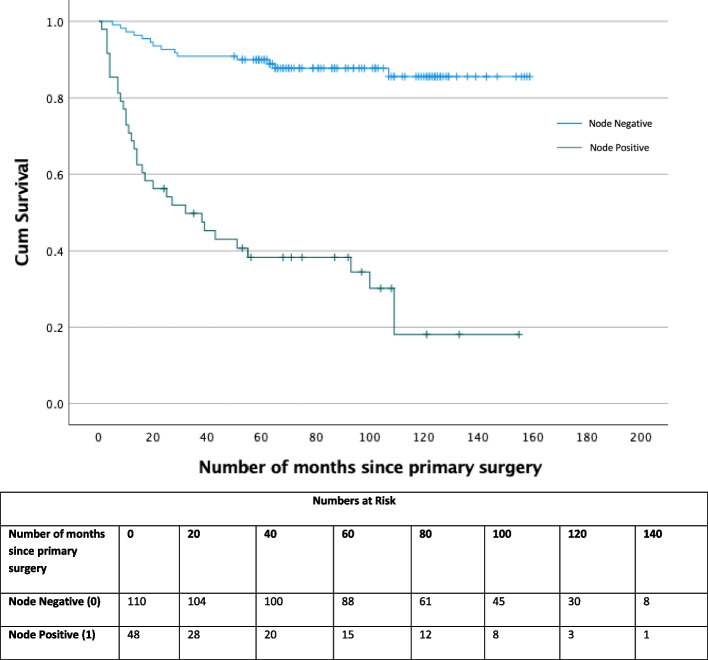
Kaplan Meier curve of Overall survival between node negative and node positive patients for all lymph node staging procedures

**Fig. 4:**
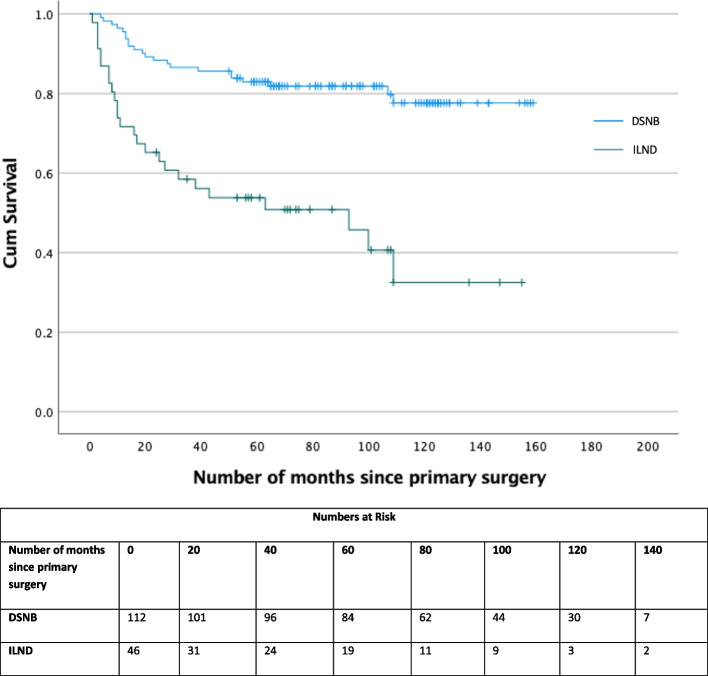
Kaplan Meier curve of Overall survival between all DSNB and ILND patients

**Fig. 5:**
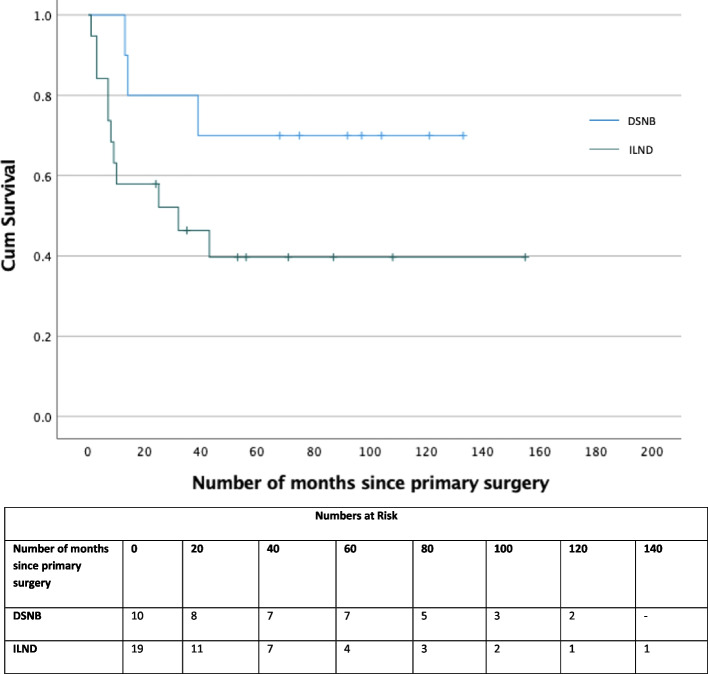
Kaplan Meier curve of Cancer Specific Survival between all node positive DSNB and ILND patients

The overall survival (OS) of patients with pN0 and pN + disease is 119 and 54 months, respectively (Fig. 3). There was also a difference in overall survival depending on which staging investigation was done at the outset (i.e., DSNB vs ILND (108 vs 72 months, *p* < 0.0001, Fig. 4)). Further pN + patients identified by DSNB (89 months) had no significant difference in cancer-specific survival compared to direct ILND patients (92 months, *p* = 0.124, Fig. 5). To our knowledge this is the first paper to report survival data in all lymph node staging procedure in penile cancer.

One patient has had a primary site recurrence of G3, pT1 SCC disease at 28 months following DSNB. His ILND after recurrence showed node-positive disease. We do not consider this patient as false-negative, as the nodal metastasis was certainly from the new primary site recurrence as per the definition in previously published literature [8]. Serial US during 28 months of initial follow-up did not show any enlarged lymph nodes. He is alive and recurrence-free to date with 55 months of total follow up.

## Discussion

Our study reports the outcome of DSNB in a contemporary era of liberal cross-sectional imaging. 30.3% of all patients had lymph node metastasis. There is no difference in mean cancer-specific survival between DSNB and ILND patients (85 vs 92 months, *p* = 0.124, log rank test) with lymph node-positive disease. To our knowledge, this is the first study from a large tertiary referral centre to report the survival and diagnostic performance on all patients who have had lymph node surgery (i.e., DSNB and ILND) with curative intent in penile cancer.

Five studies have reported results of DSNB in more than 100 patients with adequate follow-up [6–8, 10, 12]. Their DSNB lymph node positive rates were quite variable, from 4.9% to 22.3% of patients. Our result of 11.2% is in line with these studies. To our knowledge, published series on DSNB do not report the outcome of cN + patients, which is essential to ascertain the validity of such protocol [6–9]. For example, a protocol with 5% FNR might have had lower cN + pathological positive rate in ILND as opposed to protocol with 10% FNR. Ours is the first paper to report the outcomes from both cN + and cN0 patients, to provide a comprehensive strategy to manage all penile cancer patients and it can act as a benchmark for future studies. The Amsterdam approach [12] has used SPECT CT to circumvent the difficulties in using US FNAC alone for imaging. However, in our experience we find that the resolution of CT in SPECT imaging is inferior to conventional CT or MRI.

DSNB had been established as a gold standard in management of cN0 disease as ILND, as a diagnostic test, has unacceptable short- and long-term complication rates. Most studies report a false-negative rate of DSNB between 5–13%, having used clinical examination of palpability to stratify patients into cN0 and cN + disease. However, it suffers from considerable inter-observer variation and is particularly unreliable in obese patients. We also know SN localisation does not work well in the presence of enlarged nodes full of tumour or if there is infection. Subjecting the latter group of patients after negative US FNAC to DSNB may not be reliable. Hence, we had a different approach and assessed the stratification of patients into cN0 and cN + disease groups using cross-sectional imaging and proceeding to up-front VEILND (Video endoscopic Inguinal lymph node dissection), if equivocal, to address this issue. Combined clinical and radiological stratification identifies abnormal lymph nodes, as opposed to enlarged nodes. Hence, we believe that this stratification approach helped us to reduce the false-negative rate substantially with acceptable higher ILND rate. Further all the DSNBs were performed at primary procedure along with primary penile cancer surgery. This might have contributed to the excellent FNR though there are no studies to support such hypothesis.

Open ILND had been found to have a high short- and long-term complication rate of 30–70%, even in the contemporary era [13, 14]. The fear of an unacceptable complication rate has forced clinicians to resort to DSNB as a diagnostic tool in borderline cases of node enlargement. However, the advent of newer techniques, like VEILND and RAILND (Robotic assisted endoscopic inguinal lymph node dissection), with substantially reduced complication rates, has made ILND a viable alternative for DSNB in borderline cases. 22/25 cN + patients in this series had VEILND and the results have already been published [11]. Hence, our newer approach of clinical-radiological stratification and VEILND for borderline cases has offered the best approach to deal with lymph node metastasis for penile cancer patients.

In our series, DSNB is a highly reliable test with sensitivity of 100%. Previous study has explored novel technique of combining DSNB with FDG PET scanning to reduce false-negative rate and the authors reported a FNR of 5.6% and 100% mortality in those 2 FN patients [15]. Hence there is a quest to improve false negative rate in penile cancer lymph node staging. Centralisation of penile cancer services with an individualised medicine approach over a protocol-based approach and the judicious use of available radiological imaging will help to improve the outcome in this disadvantaged group of patients.

Our study has some limitations. This is a non-randomised series retrospective analysis from a single centre where most decisions are made by single supra-network MDT. Hence the stratification methodology needs to be validated in other centres. Our centre also offers VEILND/ RAILND to all the patients who require inguinal lymph node dissection with considerably reduced morbidity. As minimally invasive inguinal lymph node dissection may not be available in all centres, stratification of patients to cN + may lead to higher ILND rates and higher morbidity in those centres. Hence, subjecting patients to highly morbid open ILND to reduce false-negative rate of 10% needs to be approached with caution.

EAU guidelines recommend DSNB for patients with only intermediate (G2pT1 SCC with lympho-vascular invasion) and high risk disease (G3 with any pT or ≥ T2 disease). We performed DSNB on all patients with invasive penile cancer including low risk disease for multiple reasons. Our regional unpublished data prior to this study showed 8% LN metastasis in low-risk disease. Jakobsen et al. also showed that 6% of their low-risk cohort had lymph node-positive disease [7]. Further, we performed DSNB at the time of primary surgery as a standard to avoid multiple general anaesthetics and hospital visits. A significant proportion of these patients have not had a biopsy as the cancer was diagnosed on clinical grounds and it is difficult to be certain about the grade and stage of disease at presentation for risk stratification. In addition, 5 of the 10 low-risk patients had T1 disease at incisional biopsy and large tumours. Eventually they turned out to be G1pT1 disease though there was a clinical prediction for it to be intermediate or even high-risk, disease. None of our 10 patients suffered additional complications due to DSNB.

As previous studies have shown that most recurrences occur within 24 months, we consider this follow up of 24–150 months is adequate to detect any false-negative cases. We have also reported the harder end point of cancer-specific survival (CSS) which is similar between DSNB and ILND patients with node-positive disease.

## Conclusion

We have shown that standardisation of ultrasound reporting and use of cross-sectional imaging results to stratify patients to cN0 and cN + disease improves the outcome of lymph node staging procedures. We also report comprehensive data of all staging lymph node surgery in penile cancer for the first time, with overall lymph node metastasis rate of 30.3%. We also for the first-time report mean overall survival of 85.6% and CSS of 100% in pN0 patients following DSNB. Further large scale and randomised studies are needed to validate our results in different patient populations.

## Data Availability

Not applicable.

## Abbreviations

CSS: Cancer Specific Survival
CT: Computerised Tomography
cN0: Clinical Node negative
cN +: Clinical Node Positive
DSNB: Dynamic Sentinel Node Biopsy
FNAC: Fine Needle Aspiration Cytology
ILND: Inguinal Lymph Node Dissection
IB: Inguinal Basins
MRI: Magnetic Resonance Imaging
RAILND: Robotic Assisted Inguinal Lymph Node Dissection
SCC: Squamous Cell Carcinoma
VEILND: Video Endoscopic Inguinal Lymph Node Dissection
